# MicroRNAs in *Vitis vinifera* cv. Chardonnay Are Differentially Expressed in Response to *Diaporthe* Species

**DOI:** 10.3390/genes10110905

**Published:** 2019-11-07

**Authors:** Ales Eichmeier, Tomas Kiss, Eliska Penazova, Jakub Pecenka, Akila Berraf-Tebbal, Miroslav Baranek, Robert Pokluda, Jana Cechova, David Gramaje, Dariusz Grzebelus

**Affiliations:** 1 Faculty of Horticulture, Mendeleum-Institute of Genetics, Mendel University in Brno, Valticka 334, 69144 Lednice, Czech Republic; tomas.kiss@mendelu.cz (T.K.); eliska.penazova@mendelu.cz (E.P.); jakub.pecenka@mendelu.cz (J.P.); berraf.a@hotmail.fr (A.B.-T.); miroslav.baranek@mendelu.cz (M.B.); robert.pokluda@mendelu.cz (R.P.); jana.cechova@mendelu.cz (J.C.); d.grzebelus@ogr.ur.krakow.pl (D.G.); 2 Instituto de Ciencias de la Vid y del Vino (ICVV), Consejo Superior de Investigaciones Científicas—Universidad de la Rioja—Gobierno de La Rioja, Ctra. de Burgos Km. 6, 26007 Logroño, Spain; david.gramaje@icvv.es; 3 Department of Plant Biology and Biotechnology, Faculty of Biotechnology and Horticulture, University of Agriculture in Krakow, 31425 Krakow, Poland

**Keywords:** high-throughput sequencing, grapevine, RT-qPCR, miRNA

## Abstract

*Diaporthe* species are important pathogens, saprobes, and endophytes on grapevines. Several species are known, either as agents of pre- or post-harvest infections, as causal agents of many relevant diseases, including swelling arm, trunk cankers, leaf spots, root and fruit rots, wilts, and cane bleaching. A growing body of evidence exists that a class of small non-coding endogenous RNAs, known as microRNAs (miRNAs), play an important role in post-transcriptional gene regulation, during plant development and responses to biotic and abiotic stresses. In this study, we explored differentially expressed miRNAs in response to *Diaporthe eres* and *Diaporthe bohemiae* infection in *Vitis vinifera* cv. Chardonnay under in vitro conditions. We used computational methods to predict putative miRNA targets in order to explore the involvement of possible pathogen response pathways. We identified 136 known and 41 new miRNA sequence variants, likely generated through post-transcriptional modifications. In the *Diaporthe eres* treatment, 61 known and 17 new miRNAs were identified while in the *Diaporthe bohemiae* treatment, 101 known and 21 new miRNAs were revealed. Our results contribute to further understanding the role miRNAs play during plant pathogenesis, which is possibly crucial in understanding disease symptom development in grapevines infected by *D. eres* and *D. bohemiae*.

## 1. Introduction

The genus *Diaporthe* (Sordariomycetes, Diaporthales, Diaporthaceae) is an extremely diverse and important group of fungi. It was proposed by Nitschke in 1870, with *Diaporthe eres* as the type species. This genus includes species that are pathogens, endophytes, and saprobes on hundreds of host plants, comprising agricultural crops, ornamental plants, and fruit and forest trees [1,2,3,4]. *Diaporthe* species are considered causal agents of grapevine trunk diseases [5]. Several species are well-known pathogens worldwide and are responsible for losses on a broad range of plants and economically important crops, including almond, apple, camellia, citrus, cucurbits, eggplant, grapevine, sunflower, peach, pear, persea, plum, soybean, and cranberries [3,6,7,8,9,10,11,12,13,14,15,16,17,18].

Numerous species have been described as causal agents of pre- or post-harvest infections. They are responsible for different disease symptoms, such as swelling arm, trunk cankers, leaf spots, rots, wilts, and cane bleeding [3,19,20,21,22,23]. Interestingly, many *Diaporthe* species can occur at the same time on diverse hosts or even on the same host or lesion [19,21,24,25].

Species in this genus are important pathogens of grapevine, causing cankers and other dieback symptoms in all major viticulture regions worldwide [24,26]. Several studies reported *D. ampelina* (=*Phomopsis viticola*) as the main species associated with Phomopsis cane and leaf spot all over the world. However, other *Diaporthe* species also have the ability to produce severe symptoms on grapevine, including *D. eres, D. ambigua*, *D. foeniculina*, *D. amygdali*, *D. australafricana*, *D. baccae*, *D. celeris*, *D. eres*, *D. foeniculina* (as *D. neotheicola*), *D. helianthi*, *D. hispaniae*, *D. hongkongensis*, *D. hungariae*, *D. kyushuensis*, *D. perjuncta*, *D. phaseolorum*, *D. rudis*, and *D. sojae* [4,24,26,27]. Their pathogenicity on grapevine has been confirmed on detached shoots, with a high variability in virulence [4,19,27]. However, certain environmental factors may accentuate or reduce the pathogenicity of the fungi. In addition, a high diversity of *Diaporthe* species observed in diseased vines does not exclude the possibility of a synergistic action of several species in causing disease. Moreover, this variability in virulence could be explained by the fact that plants produce many secondary metabolites, some of which have antimicrobial properties and may protect the plant against attacks [28]. For instance, grapevine develops various mechanisms at a physiological and molecular level in order to cope with the difficulties with biotic and abiotic factors in their environment [29]. Important progress has been made to understand plant–pathogen interactions and the multiple gene regulatory systems that they use during plant defense responses. Axenic cultivation of *Diaporthe* spp. allows direct in vivo investigation of molecular interactions postulated to exist between *Diaporthe* spp. and their plant hosts [14]. Additionally, high-throughput sequencing (HTS) of transcriptomes, as well as proteomics, has served as a valuable approach to gain new insights into physiological, biochemical, and molecular mechanisms underlying *Diaporthe* spp. disease symptom development in other plant species, such as asperge (*Asparagus* spp.) or rice [30,31,32].

A class of small non-coding endogenous RNAs known as microRNAs (miRNAs) plays a major role in post-transcriptional gene regulation during plant development and plant responses to biotic and abiotic stresses [33,34]. Mature miRNAs are typically 19 to 24 nt in length and originate from miRNA (MIR) genes that are transcribed by RNA polymerase II. The transcripts, known as primary miRNAs (pri-miRNA), form imperfect fold-back hairpins that are cleaved by RNase III-like Dicer 1 (DCL1) to produce miRNA precursors (pre-miRNA). Each pre-miRNA contains one or more short intermediate complementary miRNA/miRNA duplexes [35]. These duplexes are then cleaved by DCL1 from the stem region and processed inside the nucleus to be exported to the cytoplasm, where the leading miRNA is incorporated into the RNA-induced silencing complex (RISC). When associated with RISC, guided binding of the miRNA to its complementary target mRNA(s) or non-coding trans-acting siRNA (TAS) transcript(s) occurs [33]. This facilitates either translational inhibition or degradation of target mRNA(s) or slicing of TAS transcripts that leads to generation of trans-acting siRNAs (tasiRNAs). Target degradation occurs through endonucleolytic cleavage by the RISC core protein Argonaute 1 (AGO1) [36,37,38]. The mechanism of RNA silencing in plants is also used in advanced detection techniques of viruses [39].

It has been suggested that the miRNA pathway contributes to pathogen-associated molecular pattern (PAMP)-triggered immunity (PTI), which refers to a basal defense response upon recognition of certain pathogenic elements. To date, the miRNA defense responses in *Arabidopsis*, rice, and a broad plant host range infected by pathogenic fungus have been evaluated [40,41,42], but none of these studies have been performed on the interaction of grapevine–trunk disease (GTD) pathogens.

The availability of two drafts of *V. vinifera* cv. Pinot Noir genome sequences obtained from high-throughput data [43,44] has enabled rapid discovery of miRNAs that further supports the efforts to explore small RNA (sRNA)-based regulatory networks in grapevine. Computational analyses of high-throughput sequencing data, followed by experimental validation, have been used to identify highly conserved miRNAs, some of which play important roles in grapevine development [45,46]. To date, 186 mature grapevine miRNA sequences from 47 different miRNA families have been deposited in miRbase: the microRNA database [47].

Here, we hypothesized that *D. eres*, as a well-known GTD pathogen, would trigger a strong response of the miRNA machinery while *D. bohemiae*, described as non-pathogenic on grapevine, would not markedly enhance the expression of miRNAs associated with disease symptoms. We used computational resources for the in silico prediction and annotation of putative miRNA targets to explore the involvement of possible pathogen response pathways. An understanding the sRNA-mediated gene regulation may be crucial to the understanding of gene regulatory pathways involved in a range of stress-regulated physiological processes. Our results provide insight into miRNA-mediated pathogenesis in *V. vinifera* and may uncover new disease control strategies for molecular breeding.

## 2. Materials and Methods

### 2.1. Plant Material

Cultivar Chardonnay clone CHAR PO-156/4 was used in this study. Shoots were sampled at the end of the growing season in 2018 and buds with meristems were used for in vitro micropropagation. In vitro cultures were established using nodal segments grown on the Murashige and Skoog medium containing 1.33 μM 6-benzylaminopurine (BA) and 0.57 μM indole-3-acetic acid (IAA). The cultures were maintained at 23 °C with a 16/8 h cycle of light and dark. The experimental plantlets were transferred to a fresh medium after three weeks. Each plantlet was placed into a separate cultivation vessel. Six-week-old cultures were rooted on the MS medium with 0.81 μM naphtalene acetic acid (NAA) [48]. We used in vitro plants because we hypothesized that a less influenced environment would be reached by using controlled abiotic and biotic factors compared to in vivo.

### 2.2. Fungal Isolates

Single-spore isolates of *D. bohemiae* strain CBS 143347 and *D. eres* strain CPC 28220 were used in this study. These two species were isolated from grapevine showing GTD symptoms in the Czech Republic [4]. The isolates were placed on potato dextrose agar (PDA) and cultivated for 10 days at 25 °C in the dark.

### 2.3. Plant Inoculation

Trials were conducted on six-week-old rooted vines. Leaves of plantlets were inoculated with a 3-mm plug of 10-day-old cultures of either *D. bohemiae* (DB) or *D. eres* (DE) using sterile plastic tips. Leaves of control plants (C) were inoculated with uncolonized sterile PDA plugs. One leaf per plant was inoculated and five plants per treatment were used. The experiment was repeated after two weeks.

### 2.4. RNA Extraction and Quality Control

RNAs were extracted from all inoculated and control plants 10 days after inoculation using PureLink™ Plant RNA Reagent (Thermo Fisher Scientific, Waltham, MA, USA), according to the manufacturer‘s instructions. The total RNA yield and quality were measured using a Bioanalyzer 2100 (Agilent Technologies, Palo Alto, CA, USA) using the Agilent RNA 6000 Nano Kit and Modulus™ Single Tube Multimode Reader (Turner Biosystems, Sunnyvale, CA, USA) using the Quant-iT™ RNA Assay Kit (Thermo Fisher Scientific, Waltham, MA, USA). Samples with an RNA Integrity Number (RIN) below 7 were excluded from further analysis. Only RNA concentrations higher than 5 ng μL^−1^ were used, and all samples at higher concentrations were diluted to 5 ng μL^−1^ based on fluorimetry. After RNA quantification, samples were pooled in groups according to the variant of inoculation, resulting in a total of five replicates per variant.

### 2.5. Library Preparation and Sequencing

A small RNA library was constructed using the TruSeq small RNA library preparation kit (Illumina, San Diego, CA, USA) and purification was done with the TailorCut Gel Extraction Tool Set (SeqMatic, Fremont, CA, USA). The quality and quantity of the library were determined using the Agilent High Sensitivity DNA Kit (Agilent, Santa Clara, CA, USA). The quantity of libraries was also determined by a Modulus™ Single Tube Multimode Reader (Turner Biosystems, Sunnyvale, CA, USA) using a Quant-iT™ dsDNA Assay Kit (Thermo Fisher Scientific, Waltham, MA, USA) and finally with a MCNext™ SYBR^®^ Fast qPCR Library Quantification Kit (MCLAB, San Francisco, CA, USA) used with Rotor-Gene 3000 (Corbett Research, Sydney, Australia). All the kits were used according to the manufacturer’s instructions. The libraries were pooled according to fluorimetry as 2 nM, supposing that the final small RNA fragments were ~150 bp.

For the sequencing run, a final pooled library of small RNAs consisted of three pooled samples/variants per one run. Sample C was labelled with index RPI5 (ACAGTG), sample DB with index RPI6 (GCCAAT), and sample DE with index RPI7 (CAGATC). The second run with repetitions also consisted of three pooled samples. Sample C was labeled with index RPI12 (CTTGTA), DB with index RPI10 (TAGCTT), and DE with index RPI11 (GGCTAC).

The libraries were sequenced with the MiniSeq instrument (Illumina, San Diego, CA, USA) using the MiniSeq High Output Reagent Kit, 75-cycles (Illumina, San Diego, CA, USA) providing 36-nt long reads.

### 2.6. Bioinformatics and Data Evaluation

The MiniSeq reads were demultiplexed using the Illumina bcl2fastq2 Conversion Software v2.20.0.422 (Illumina). The sequence quality was controlled by FastQC-0.10.1 [49]. Then, the reads were transformed to the fasta format using fastq_to_fasta (fastx-0.0.14, http://hannonlab.cshl.edu/fastx_toolkit/) and a Phred score was assigned a Q score of 30 (Q30); reads were trimmed using fastx_clipper (fastx-0.0.14), and the unique reads were obtained using fastx_collapser (fastx-0.0.14). Datasets corresponding to the same treatment were merged into one file. The total number of known miRNAs was counted and annotated using the CLC Genomics Workbench 6.5.1 (CLC Bio, Aarhus, Denmark).

### 2.7. miRNA Target Prediction and Functional Annotation

**Figure 1:**
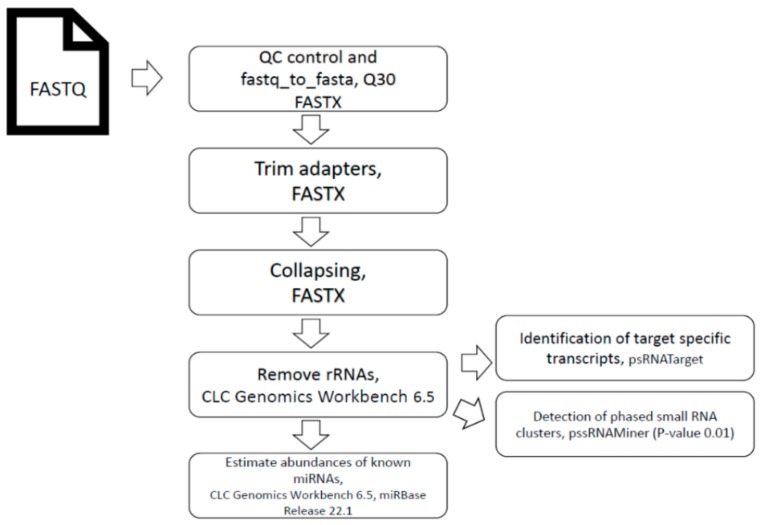
Flow chart of data processing.

The unique (non-redundant) 19 to 25 nt sequences, across all six libraries representing the three treatments in two replicates, in total included the pool of five plants per treatment. Thus, 30 grapevine plants were used in this study. Datasets were submitted to the psRNATarget Analysis server (http://plantgrn.noble.org/psRNATarget/) to predict miRNAs [50]. The pssRNAMiner web server (http://bioinfo3.noble.org/pssRNAMiner/) [51] was used to identify both the clusters of phased small RNAs as well as the potential phase initiator. The CLC Genomics Workbench 6.5.1 (CLC Bio, Aarhus, Denmark) was used to calculate the abundance of unique miRNAs, and counting of the reads was done using UNIX custom scripts. The pipeline is outlined in Figure 1.

### 2.8. Validation of miRNA Expression Profiles by Real-Time RT-qPCR

Small RNA sequencing on a MiniSeq system provided input data for the selection of sequences based on their different expression levels and prediction of fold-back structures. Stem-loop reverse transcription quantitative PCR (RT-qPCR) assays were performed according to the methods of Chen et al. [52] to validate the small RNA sequencing results. High-quality total RNA was prepared as described above. The total RNA of samples from each variant and replication were pooled equally according to the volume. Replications of each variant were then pooled according to the RNA weight. Finally, one pooled total RNA was prepared for each variant. For each miRNA, a 20-μL reverse transcription reaction was prepared containing 100 U Superscript III reverse transcriptase (Invitrogen, Carlsbad, CA, USA), 20 U RiboLock RNase inhibitor (Thermo Scientific, Waltham, MA, United States), 1× first-strand buffer, 5 mM DTT, 500 nM dNTPs, 1 μM miRNA-specific stem-loop RT primer, and 0.8 μg pooled total RNA. Reverse transcription cycling conditions were as follows: 30 min at 16 °C, 60 min at 42 °C, and heat inactivation for 10 min at 75 °C. qPCR was performed using the Universal ProbeLibrary (UPL) probe assay with UPL probe #21 (Roche Diagnostics, Basel, Switzerland). Each 10 μL of reaction mixture was prepared in triplicate and contained 1 μL cDNA, 1× Colorless GoTaq Reaction Buffer (Promega, Madison, WI, USA), 2 mM MgCl_2_ (Promega), 1 U GoTaq G2 DNA Polymerase (Promega), 0.5 μM miRNA-specific forward primer, 0.5 μM universal reverse primer, 0.2 μM UPL probe, and nuclease-free water. The primer sequences are provided in Table S1. A control reaction, without a cDNA template, was included for each miRNA. Based on results from the geNorm analysis [53] (qBasePLUS v3.2, Biogazelle, Ghent, Belgium), miR166c was chosen as a reference to normalize miRNA expression levels. The Pfaffl method [54] was used for normalization to the reference miRNA. PCR amplification was performed in an ECO Real-Time PCR System (Illumina, San Diego, CA, USA), in which the baseline and threshold cycles (Cq) were automatically determined with Eco Real-Time PCR System Software. Cycling conditions were as follows: 95 °C for 2 min, 40 cycles at 95 °C for 15 s, and 60 °C for 1 min. Relative miRNA expression analysis was performed using qBasePLUS v3.2 software (Biogazelle, Ghent, Belgium) [55].

## 3. Results

### 3.1. Plant Inoculation

**Figure 2:**
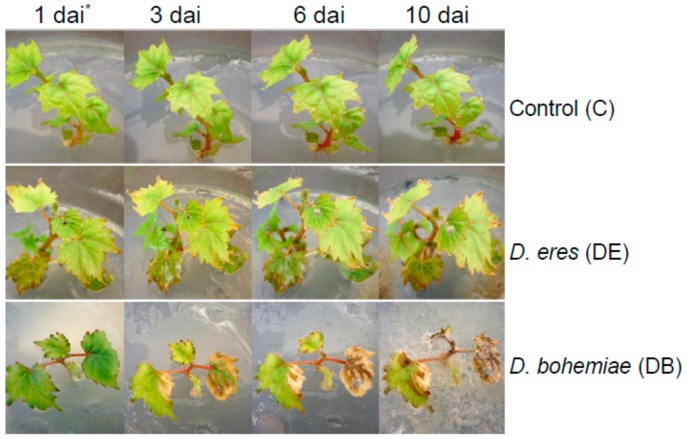
Treatments used in the study. * days after inoculation.

Plants inoculated with *D. eres* did not show any symptoms on the first, third, and sixth day after inoculation (dai). However, small lesions on the leaves appeared on 10 dai (Figure 2). In contrast, plants inoculated with *D. bohemiae* showed visible symptoms on the leaves only 3 dai (Figure 2). Subsequently, brown necrosis occurred on the leaves within 6 dai. From the 10th day, more than half of the plants died. The control plants did not develop any symptoms (Figure 2).

### 3.2. The Abundance of sRNAs in Grapevines in Vitro

**Figure 3:**
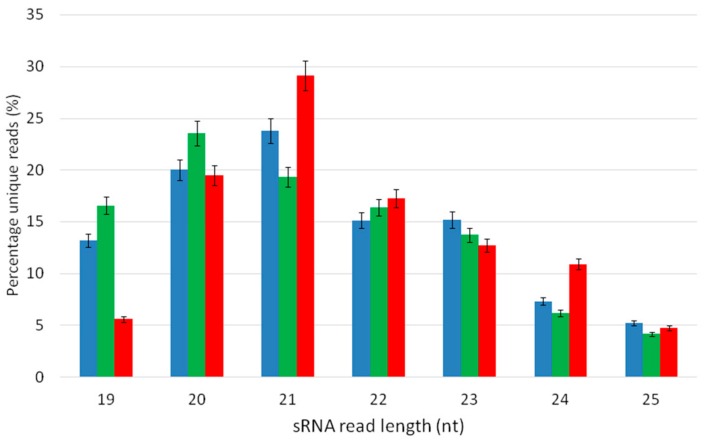
Bar plot depicting the size distribution of unique reads, psRNATarget. Blue—DE, green—DB, Red—C.

In the present study, libraries representative of sRNA populations extracted from grapevine treatments DE, DB, and C, and sequenced by Illumina SBS technology, contained DE = 8.3 × 10^6^, DB = 5.8 × 10^6^, and C = 4.6 × 10^6^ reads at Q30. After clipping, collapsing, and normalization per 10^6^ reads, DE, DB, and C contained 2,178,138; 3,399,118; and 1,855,572 reads, respectively. The most abundant sRNAs were the 20- (DB) and 21-nt class (DE, C) (Figure 3). The lowest abundancy was recorded in 25-nt sRNAs through all the treatments. The most plant-decaying treatment, DB, had a similar 22- and 23-nt class profile as the DE treatment and C. A balanced sRNAs read distribution was recorded regarding the 22- and 23-nt sRNAs between all three treatments (Table 1).

### 3.3. New and Conserved miRNAs Identified in Grapevine cv. Chardonnay in Vitro

**Figure 4:**
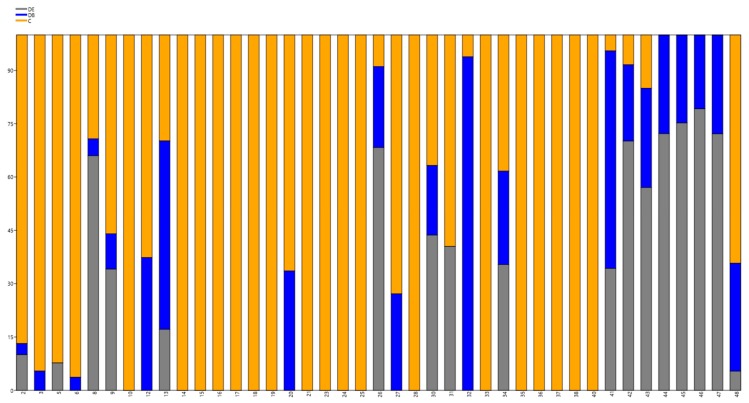
Stacked chart of normalized read counts per treatments DE, DB, and C. The plot was generated based on CLC Genomics Workbench normalized reads, generating novel small matured RNAs, and depicted using PAST version 3.25. The numbers on axis X correspond to Table 2, column miRNA name. Percentages are depicted on axis Y.

Sequence analysis coupled with the fold-back structure predictions for potential novel miRNAs led us to identify 41 new candidate miRNAs from grapevine (Table 2). Regarding the different expression levels, which are highlighted on the Figure 4, it is supposed that two miRNAs, 32 (CCCAGUCCCGAACCCGUCGG) and 41 (CCGGCGAUGCGCUCCUGGCC), are linked with the pathogenicity of DB. A gene encoding a Golgi protein [56] involved in several signaling events could be a putative target of miRNA 32. miRNA 41 is probably associated with the expression of RPP13-like protein 1, a potential disease resistance protein. Representation of the newly identified miRNAs within the treatments is presented in Table S4.

**Figure 5:**
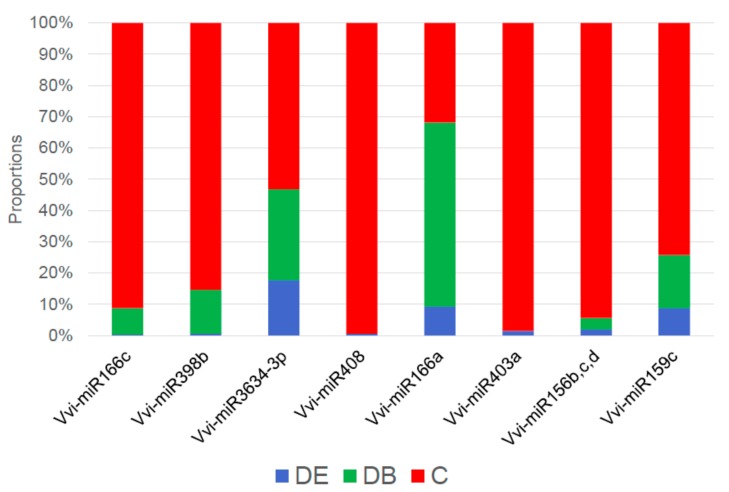
Bar plot depicting the proportions of treatments across known detected grapevine matured miRNAs, CLC Genomics Workbench, miRBase Release 22.1. Proportions were calculated based on normalized total reads.

**Figure 6:**
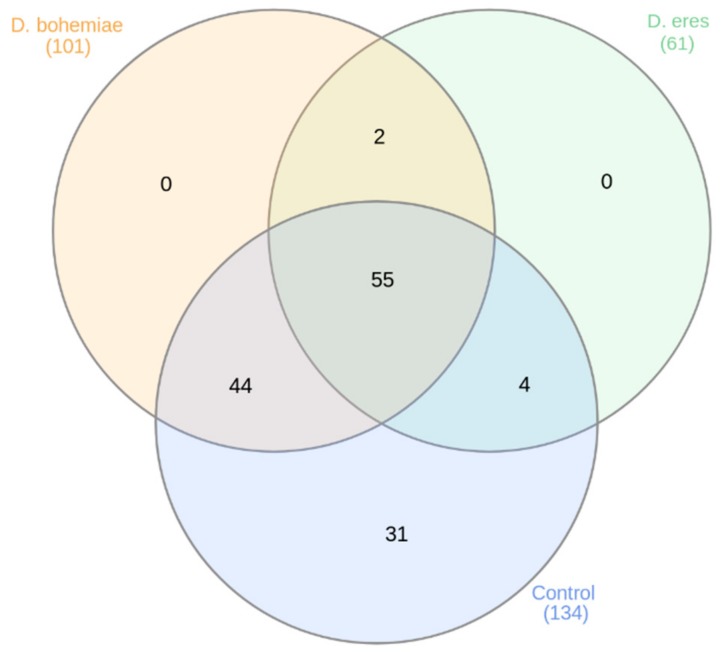
Venn diagram showing the representation of 136 known miRNAs.

A total of 136 conserved miRNAs were identified in grapevine cv. Chardonnay in vitro (Table S2). Among them, eight differentially expressed known grapevine miRNAs were revealed (Figure 5). Representation of the known miRNAs within the treatments is depicted in Figure 6, Table S3. The most abundant miRNAs were identified in the control, a more than 80% abundance in the case of Vvi-miR166c, Vvi-miR166a, Vvi-miR403a, and Vvi-miR156b,c,d. Distinctly, in the most affected DB treatment, Vvi-miR166a was the most abundant.

### 3.4. miRNA Expression Profiles by Real-Time RT-qPCR

Real-time RT-qPCR revealed the expression profiles of nine miRNAs, two novel and seven known, for grapevine (Figure S1). Both novel miRNAs, 32 and 41, were overexpressed in DB-inoculated plants and less expressed in DE and C. Their overexpression in DB was confirmed by RT-qPCR, while it also revealed a higher expression of miRNA 32 and 41 in the C than the DE treatment, which was not observed in the small RNA sequencing results (Figure 4).

Known grapevine miRNAs Vvi-miR156b,c,d, Vvi-miR166a, Vvi-miR166c, Vvi-miR3634-3p, Vvi-miR398b, Vvi-miR403a, and Vvi-miR408 were also quantified by RT-qPCR. Both miRNAs of the 166 family were highly expressed in all treatments, with a higher abundance in C and DB, as compared to DE. A high overexpression in C was revealed for miRNAs Vvi-miR156b,c,d and Vvi-miR398b, in agreement with the small RNA sequencing results. The RT-qPCR results also revealed an overexpression of miRNA Vvi-miR408 in DB, but according to small RNA sequencing, it was upregulated in C (Figure 4 and Figure 5). Similarly, Vvi-miR403a, which was most abundant in DE according to RT-qPCR, showed over a 90% abundance in C as revealed by small RNA sequencing. Vvi-miR166c and Vvi-miR3634-3p showed a relatively similar expression in all treatments in small RNA sequencing, which was further confirmed by RT-qPCR.

## 4. Discussion

This is the first attempt to use small RNA high-throughput sequencing data to identify miRNAs differentially expressed in *V. vinifera* cv. Chardonnay in response to *D. eres* and *D. bohemiae* isolated from grapevine in the Czech Republic. The experimental strategy applied in this study was designed to investigate the profile of grapevine miRNAs in response to fungal infection in vitro because there are no unpredictable abiotic factors. Plant-pathogen interactions were entirely dissociated from the environment, which is usually used in the sRNAs profiling of stressed plants [57,58]. Ma et al. [58] also found that the fungal sRNA enrichment was lower in planta than during in vitro growth. In this study, we used the cultivar Chardonnay since it is a popular grapevine cultivar all around the world [59], and in previous research, it showed a high level of tolerance against natural infections of GTD pathogens in Italy [60,61].

In this study, we revealed that the most abundant sRNAs were the 20- (DB) and 21-nt class (DE, C), corresponding with the results of Pantaleo et al. [45]. Previous studies showed that 24-nt sRNAs were more abundant in plants than the 21-nt class [62,63,64]. This is possibly because of the concerted activity of plant-specific DNA-dependent RNA polymerases, PolIVa and PolIVb, with the accumulation of 24-nt heterochromatic siRNAs via RDR2-mediated dsRNA formation and DCL3-mediated processing [65,66]. Many known grapevine miRNAs [45] were found in our datasets from whole grapevine plants cultivated in vitro (Table S2). In addition, 41 novel miRNA candidates were identified. In general, the selected miRNA profiles measured by qRT-PCR confirmed the sequencing data. A few discrepancies observed were within the range of those reported by Pantaleo et al. [67]. Similar inconsistencies were also previously reported for some miRNAs when high-throughput sequencing and northern blot analyses were compared, for example, for miR3633 and some other conserved and grapevine-specific miRNAs [45]. Small RNA high-throughput sequencing is reported to produce bias. Further, the use of different adapters and barcodes during ligation as well as complex RNA structures and modifications affect cDNA synthesis efficacies and exemplify sources of bias in deep sequencing [68]. We also observed some discrepancies in the detection of novel miRNA candidates, miRNA names 6 [69], 16 [70], 24, 36, and 38 [43] in Table 2, suggesting that these miRNAs are already known.

The miRNA candidates 32 and 41 have not been previously reported. We were able to amplify them by RT-qPCR, thus identifying their targets. For the miRNA 32 targets chr6.gff3_MRNA_VIT_06s0004g04740.t01 (Except 3.0, Inhibition-Cleavage), we found that it targets the mRNA sequence acyl-CoA-binding protein domain containing protein 3, which is a Golgi protein involved in several signaling events [56]. This could be linked with the higher in vitro virulence of *D. bohemiae* compared to *D. eres*. Golgi body-mediated signaling is linked to its fragmentation and regeneration during the mitotic cycle of the cell. During this process, Golgi-resident proteins are released to the cytosol and interact with other signaling molecules to regulate various cellular processes. Acyl-coenzyme A binding domain containing 3 protein (ACBD3) is a Golgi protein involved in several signaling events. ACBD3 protein was previously known as a peripheral-type benzodiazepine receptor and cAMP-dependent protein kinase associated protein 7 (PAP7), Golgi complex-associated protein of 60 kDa (GCP60), Golgi complex-associated protein 1 (GOCAP1), and Golgi phosphoprotein 1 (GOLPH1) [56]. If the regeneration process during the mitotic cycle of the cell is influenced by an abundancy of miRNA 32, it would be associated with a higher virulence of *D. bohemiae*.

Regarding miRNA 41 targets chr12.gff3_MRNA_VIT_12s0034g02480.t01 (Except 3.0, Inhibition-Cleavage), the miRNA 41 is focused on the mRNA sequence with an expression of RPP13-like protein 1, which is potentially a disease resistance protein. This phenomenon was described by [71], proving that RPP13 is a simple locus in *Arabidopsis thaliana* for alleles that specify downy mildew resistance to different avirulence determinants in *Peronospora parasitica*. It could be the case in our study that the miRNA 41 regulates the pathogenicity of the fungus *Diaporthe* on the grapevines in vitro. It could be the case that miRNA 41 and RPP13 are elements of the resistance mechanism [71], as RPP13 are well-known plant R genes governing hypersensitivity (HR)-based resistance [72], which may result in the phenotypic effect observed upon massive inoculation by DB in vitro, while the pathogen is effectively eliminated by grapevine in regular (field) conditions.

The results suggest some hypothetical interactions between miRNAs and the physiological changes induced in grapevine by *Diaporthe* in vitro (miR3634, miR408, miR403) similar to those described by Pantaleo et al. [67] that linked miRNAs, physiological changes, and *Grapevine rupestris stem-pitting associated virus* infection with miR156, miR164, miR319, miR394, and miR396.

Vvi-miR166c and Vvi-miR166a are thought to target mRNA coding for HD-Zip transcription factors, including Phabulosa (PHB) and Phavoluta (PHV), that regulate axillary meristem initiation and leaf development [73]. MicroRNAs 165 and 166 are able to cleave their target mRNAs of *HD-ZIP III* genes, thus regulating the functions of these genes [74]. Du et al. [75] indicated that class III homeodomain leucine zipper transcription factors (HD-ZIP III TFs) and microRNA 165/166 (miR165/166) may play important roles in secondary cell wall formation. The HD-ZIP III TFs regulate a number of developmental processes, such as embryo patterning, meristem initiation and homeostasis, lateral organ polarity, and vascular development, in *Arabidopsis* [76].

The miR166 overexpresssors exhibit an enlargement of the shoot apical meristem (SAM) and an enhancement of vascular development of *Arabidopsis* [77]. The expression level of miRNAs 166 was not linked with *Diaporthe* infection on grapevines in this study. Based on HTS analysis, Vvi-miR166c was mostly expressed in the C treatment, which corresponds to previously published research by Jung et al. [78]. This finding indicated that SAM machinery works properly in a balanced expression of miR165 and miR166; however, no differences between treatments were found by RT-qPCR [78].

In our study, the analyses of miR166a did not show clear conclusions. According to RT-qPCR, the lowest expression level was in DE and was almost similar in DB and C. On the other hand, Kim et al. [79] reported that the *men*1 mutant of *Arabidopsis* overexpressing the *MIR166a* gene exhibited pleiotropic phenotypes, such as stunted growth, disrupted floral structure, fasciated inflorescence stem, and enlarged SAM. Our HTS analysis showed the lowest expression level in DE and the highest in DB.

We analyzed these miR398 mostly overexpressed miRNAs in C by both methods. The Vvi-miR398b sequence belongs to the miR398 family of miRNAs, which are predicted to target mRNAs coding for copper superoxide dismutases an cytochrome C oxidase subunit V [80].

Our study provided data that described a similar representation of Vvi-miR3634-3p in DE, DB, and C. Chitarra et al. [81] reported a similar representation of Vvi-miR3634-3p. Vvi-miR3634-3p were identified by [45] as being up-regulated in *Grapevine rupestris stem-pitting associated* virus-infected grapevines. Vvi-miR3634-3p were also the most expressed in “Barbera” grapevine leaf midribs that were infected with *Flavescence dorée* [81].

Mica et al. [46] indicated that miRNAs Vvi-miR408 were extremely highly expressed in root tissues, targeting various copper proteins: Plantacyanin, laccases, and a superoxide dismutase, all putatively involved in stress responses and lignification. These miRNAs have also been shown to be coexpressed in *Arabidopsis* under conditions of copper deprivation [82]. Our results agree with their findings because the in vitro media had low contents of copper. *Diaporthe* infection probably influenced the Vvi-miR408 expression. HTS revealed that *Diaporthe*-infected grapevines were Vvi-miR408 downregulated but RT-qPCR showed the highest expression of these miRNAs in DB, thus there could also be a link with the biotic stress caused by fungal infection.

Vvi-miR403a encodes a miRNA that targets AGO2 and AGO3 [83]. Regarding the results of this study, Vvi-miR403a were mostly expressed in the control treatment according to HTS, but DE showed the highest expression of Vvi-miR403a according to RT-qPCR. Vvi-miR156b,c,d are predicted to target mRNAs coding for squamosa-promoter binding protein (SBP)-like transcription factors and our HTS and RT-qPCR results confirmed the highest expression in C. These genes encode a family of plant-specific transcription factors that play vital roles in plant growth and development [84].

Vvi-miR159c is usual plant miRNA and is thought to target mRNAs coding for MYB proteins that are known to bind to the promoter of the floral meristem identity gene *LEAFY* [73]. Flowering plants produce floral meristems in response to intrinsic and extrinsic flowering inductive signals [85]. According to our results, these miRNAs were also mostly expressed in the C treatment.

In general, *D. bohemiae* was more pathogenic to grapevine than *D. eres*, unlike the results of Guarnaccia et al. [4]. This can be explained by the fact that Guarnaccia et al. [4] used a different inoculation method for *Diaporthe* [19,27], using green shoots cut from healthy mature grapevine cv. Riesling, and the shoots were artificially inoculated with a 1-week-old 6-mm agar plug to determine the pathogenicity. Here, we used a 1-week-old 3-mm plug for the inoculation of a single leave of grapevine cv. Chardonnay in vitro; this was repeated two times, and the pathogenicity to the grapevine was confirmed in vitro. Additionally, the life cycle of *Diaporthe* on grapevines starts on green parts and more intensively on the leaves [86]. The pathogenicity of *D. bohemiae* CBS 143347 should be further studied with different methods of inoculation.

## 5. Conclusions

The outcomes of this study provide novel insights into *D. eres* and *D. bohemiae* pathogenicity and the *V. vinifera* cv. Chardonnay defense mechanism in vitro. The results revealed that *D. eres* has the ability to be phytopathogenic and that it triggers some specific miRNAs expression. Surprisingly, *D. bohemiae* was previously published as non-phytopathogenic fungus, but in our study, it was found to be more virulent than *D. eres*. In addition, some selected miRNAs were expressed more in *D. bohemiae* than in both the *D. eres* and control treatments. We also identified two novel miRNAs, named 32 and 41, which appear to be linked with the pathogenicity of *D. bohemiae* in vitro. Further studies focusing on the mechanism of RNA silencing, used as a strategy against grapevine trunk disease pathogens, are necessary to understand the mechanism of fast, strong, and effective defense responses to grapevine trunk fungal pathogens.

## Figures and Tables

**Table 1:** Numbers of size distributions of unique reads normalized per 1,000,000.

	19	20	21	22	23	24	25	Total
DE	287,897	436,557	518,754	330,042	331,108	159,806	113,975	2,178,138
DB	563,942	801,579	656,600	556,699	467,013	211,409	141,876	3,399,118
C	104,300	362,066	540,728	320,848	236,014	202,736	88,881	1,855,572

**Table 2:** Putative miRNAs identified using CLC Genomics WB 6.5.1, putative targets determined by blastN/NCBI and by psRNATarget with the possible type of inhibition.

miRNA Name	miRNA Sequence	Putative Target Identified Using NCBI	Target Acc. Based on the Highest Expectations (E), psRNA Target	Inhibition
2	CCCAGUCCCGAACCCGUCGGC	similar to aspartate aminotransferase; similar to Aspartate aminotransferase 2, transcript variant X9, misc_RNA, importin α isoform 9	chr11.gff3_MRNA_VIT_11s0149g00200.t01	Cleavage
3	AGUUACUAAUUCAUGAUCUGGC	importin α isoform 9	chr2.gff3_MRNA_VIT_02s0033g00980.t01	Cleavage
5	CCAGUCCCGAACCCGUCGGC	Vitis vinifera contig VV78X128415.10, whole genome shotgun sequence	chr7_random.gff3_MRNA_VIT_07s0151g00980.t01	Cleavage
6	UCUCGGACCAGGCUUCAUUCC	Vitis vinifera microRNA MIR166a (MIR166A), microRNA, http://www.mirbase.org/cgi-bin/mature.pl?mature_acc=MIMAT0020658	chr18.gff3_MRNA_VIT_18s0075g00480.t01	Translocation
8	GGUGGCUGUAGUUUAGUGGU	Vitis vinifera contig VV78X038801.3, whole genome shotgun sequence	chr18.gff3_MRNA_VIT_18s0001g12770.t01	Cleavage
9	CGGUGGACUGCUCGAGCUGC	Vitis vinifera contig VV78X196950.19, whole genome shotgun sequence	chr15.gff3_MRNA_VIT_15s0048g02810.t01	Translocation
10	CUAACAGACCGGUAGACUUGAAC	Vitis vinifera contig VV78X130314.7, whole genome shotgun sequence	chr15.gff3_MRNA_VIT_15s0048g02810.t01	Translation
12	CCCAGUCCCGAACCCGUCGGCU	Vitis vinifera contig VV78X156561.10, whole genome shotgun sequence	chr11.gff3_MRNA_VIT_11s0149g00200.t01	Cleavage
13	GCGCCUGUAGCUCAGUGGA	Vitis vinifera contig VV78X046944.3, whole genome shotgun sequence	chr8.gff3_MRNA_VIT_08s0007g07620.t01	Cleavage
14	UUCAUGGACGUUGAUAAGAUCCU	Vitis vinifera subsp. sylvestris chloroplast DNA, complete genome	chr7.gff3_MRNA_VIT_07s0005g00750.t01	Cleavage
15	UAACAGACCGGUAGACUUGAAC	PREDICTED: Vitis vinifera pentatricopeptide repeat-containing protein At5g50990 (LOC100247459)	chr18.gff3_MRNA_VIT_18s0001g09480.t01	Cleavage
16	UGCACUGCCUCUUCCCUGGCU	Vitis vinifera microRNA MIR408 gene, complete sequence, http://www.mirbase.org/cgi-bin/mirna_entry.pl?acc=MI0005917	chr18.gff3_MRNA_VIT_18s0001g15240.t01	Cleavage
17	CCUAACAGACCGGUAGACUUGAAC	PREDICTED: Vitis vinifera ATP synthase subunit α, chloroplastic-like (LOC109124299), mRNA	chr18.gff3_MRNA_VIT_18s0001g11300.t01	Cleavage
18	UCCUAACAGACCGGUAGACUUGAAC	Vitis vinifera subsp. sylvestris chloroplast DNA, complete genome	chr18.gff3_MRNA_VIT_18s0001g11300.t01	Cleavage
19	UCCUAACAGACCGGUAGACUUGAAC	Vitis vinifera subsp. sylvestris chloroplast DNA, complete genome	chr18.gff3_MRNA_VIT_18s0001g11300.t01	Cleavage
20	UUAGAUGAUCAUCAACAAACU	Vitis vinifera ankyrin repeat-containing protein NPR4-like (LOC100260982), transcript variant X10, mRNA	chr7.gff3_MRNA_VIT_07s0005g02430.t01	Cleavage
21	CAGACCGGUAGACUUGAAC	PREDICTED: Vitis vinifera ATP synthase subunit α, chloroplastic-like (LOC109124299), mRNA	chr19.gff3_MRNA_VIT_19s0090g01480.t01	Cleavage
23	ACAGACCGGUAGACUUGAAC	PREDICTED: Vitis vinifera ATP synthase subunit α, chloroplastic-like (LOC109124299), mRNA	chr18.gff3_MRNA_VIT_18s0001g09480.t01	Cleavage
24	UUCCACAGCUUUCUUGAACUU	Vitis vinifera microRNA MIR396b (MIR396B), microRNA	chr15.gff3_MRNA_VIT_15s0021g02580.t01	Cleavage
25	AACAGACCGGUAGACUUGAAC	PREDICTED: Vitis vinifera ATP synthase subunit α, chloroplastic-like (LOC109124299), mRNA	chr18.gff3_MRNA_VIT_18s0001g09480.t01	Cleavage
26	CCGGCGAUGCGCUCCUGGCC	Vitis vinifera contig VV78X197078.6, whole genome shotgun sequence	chr12.gff3_MRNA_VIT_12s0034g02480.t01	Cleavage
27	CAGUCCCGAACCCGUCGGC	Vitis vinifera contig VV78X156561.10, whole genome shotgun sequence	chr11.gff3_MRNA_VIT_11s0149g00200.t01	Cleavage
28	UGUUGAGCUCACCUUGUACCC	PREDICTED: Vitis vinifera kinase-interacting family protein (LOC100246194), transcript variant X1, mRNA	chr9.gff3_MRNA_VIT_09s0002g03120.t01	Translation
30	CGGUGGACUGCUCGAGCUGCU	Vitis vinifera contig VV78X156561.10, whole genome shotgun sequence	chr15.gff3_MRNA_VIT_15s0048g02810.t01	Translation
31	GUUGAGCUCACCUUGUACCCA	PREDICTED: Vitis vinifera kinase-interacting family protein (LOC100246194), transcript variant X1, mRNA	chr9.gff3_MRNA_VIT_09s0002g03120.t01	Translation
32	CCCAGUCCCGAACCCGUCGG	Vitis vinifera contig VV78X128415.10, whole genome shotgun sequence, mRNA sequence acyl CoA binding protein domain containing protein 3 which is a Golgi protein involved in several signalling events	chr6.gff3_MRNA_VIT_06s0004g04740.t01	Cleavage
33	UGAAGGUCCAAGGCCGAGGCU	PREDICTED: Vitis vinifera uncharacterized LOC100855078 (LOC100855078), ncRNA	chr14.gff3_MRNA_VIT_14s0006g03100.t01	Cleavage
34	GGGAUGGGUCGACCGGUCC	Vitis vinifera contig VV78X071755.8, whole genome shotgun sequence	chr12.gff3_MRNA_VIT_12s0034g01520.t01	Cleavage
35	UCGGAUAAAGGGUUAUACAUC	PREDICTED: Vitis vinifera uncharacterized LOC100853315 (LOC100853315), transcript variant X1, ncRNA	chr6.gff3_MRNA_VIT_06s0009g03800.t01	Cleavage
36	UGCACUGCCUCUUCCCUGGC	Vitis vinifera microRNA MIR408 (MIR408), microRNA	chr18.gff3_MRNA_VIT_18s0001g15240.t01	Cleavage
37	CUGGAUUAUGACUGAACGCCU	PREDICTED: Vitis vinifera lysosomal Pro-X carboxypeptidase (LOC100244772), transcript variant X2, mRNA	chr4.gff3_MRNA_VIT_04s0210g00160.t01	Cleavage
38	UUCCACAGCUUUCUUGAACU	Vitis vinifera microRNA MIR396c (MIR396C), microRNA	chr15.gff3_MRNA_VIT_15s0021g02580.t01	Cleavage
40	AGUUACUAAUUCAUGAUCUGGCC	PREDICTED: Vitis vinifera scopoletin glucosyltransferase (LOC100260498), mRNA	chr2.gff3_MRNA_VIT_02s0033g00980.t01	Cleavage
41	CCGGCGAUGCGCUCCUGGCC	mRNA sequence with expression of RPP13-like protein 1, potential disease resistance protein	chr12.gff3_MRNA_VIT_12s0034g02480.t01	Cleavage
42	CCGGCGAUGCGCUCCUGGCCU	PREDICTED: Vitis vinifera putative disease resistance RPP13-like protein 1 (LOC100258269), transcript variant X4, mRNA	chr12.gff3_MRNA_VIT_12s0034g02480.t01	Cleavage
43	ACCGGCGAUGCGCUCCUGGCCU	PREDICTED: Vitis vinifera auxin efflux carrier component 3 (LOC100268124), mRNA	chr1.gff3_MRNA_VIT_01s0011g01820.t01	Cleavage
44	GCCCGUGGAGACGUCGUCGCCUCG	PREDICTED: Vitis vinifera oxalate--CoA ligase (LOC100256632), mRNA	chr1.gff3_MRNA_VIT_01s0011g00770.t01	Cleavage
45	CGCCGUCCGAAUUGUAGUCUGGA	PREDICTED: Vitis vinifera uncharacterized LOC109123385 (LOC109123385), mRNA	chr12.gff3_MRNA_VIT_12s0134g00450.t01	Cleavage
46	UCGGGUUAACAUUCCUGAACCGGGA	PREDICTED: Vitis vinifera AUGMIN subunit 7 (LOC100243653), transcript variant X1, mRNA	chr1.gff3_MRNA_VIT_01s0011g01130.t01	Cleavage
47	CGGUGGACUGCUCGAGCUGCU	PREDICTED: Vitis vinifera non-specific lipid-transfer protein-like protein At5g64080 (LOC100247017), mRNA	chr15.gff3_MRNA_VIT_15s0048g02810.t01	Translation
48	CCAGUCCCGAACCCGUCGGC	PREDICTED: Vitis vinifera acyl-CoA-binding domain-containing protein 3 (LOC100268114), transcript variant X6, mRNA	chr7_random.gff3_MRNA_VIT_07s0151g00980.t01	Cleavage

## Supplementary Materials

The following are available online at https://www.mdpi.com/2073-4425/10/11/905/s1. Figure S1: Relative quantification based on stem-loop RT-qPCR of differently expressed miRNAs in different variants. miRNA Vvi-miR 166c is the reference miRNA and the bars represent fold expressions compared to the reference miRNA. DE is the *Diaporthe eres* treated, DB is *Diaporthe bohemiae* treated and C is control. Relative miRNA quantification was analysed in qBasePLUS v3.2 software (Biogazelle, Ghent, Belgium), Table S1: List of primer sequences used in the real-time RT-qPCR assay for relative quantification of target miRNAs. Table S2: Identification of total known grapevine miRNAs. Data were generated by CLC Genomics WB 6.5.1, the reads were analyzed using extraction and counting of the reads. Annotation and merging of the counts were used. Table S3: Representation of known miRNAs in the treatments DE, DB and C. Table S4: Representation of new miRNAs in the treatments DE, DB and C.
