# Supplementary material for: MicroRNAs in Vitis vinifera cv. Chardonnay Are Differentially Expressed in Response to Diaporthe Species
Source: Genes (Basel). 2019 Nov 7;10(11):905. doi: 10.3390/genes10110905 (PMC6896114; doi:10.3390/genes10110905)
Supplement: Supplementary file 1 [file genes-10-00905-s001.pdf]

Supplementary Material

MicroRNAs in *Vitis vinifera* cv. Chardonnay are differentially expressed in response to *Diaporthe* spp.

Ales Eichmeier*, Tomas Kiss, Eliska Penazova, Jakub Pecenka, Akila Berraf-Tebbal, Miroslav Baranek, Robert Pokluda, Jana Cechova, David Gramaje, Dariusz Grzebelus

***Correspondence:** ales.eichmeier@mendelu.cz

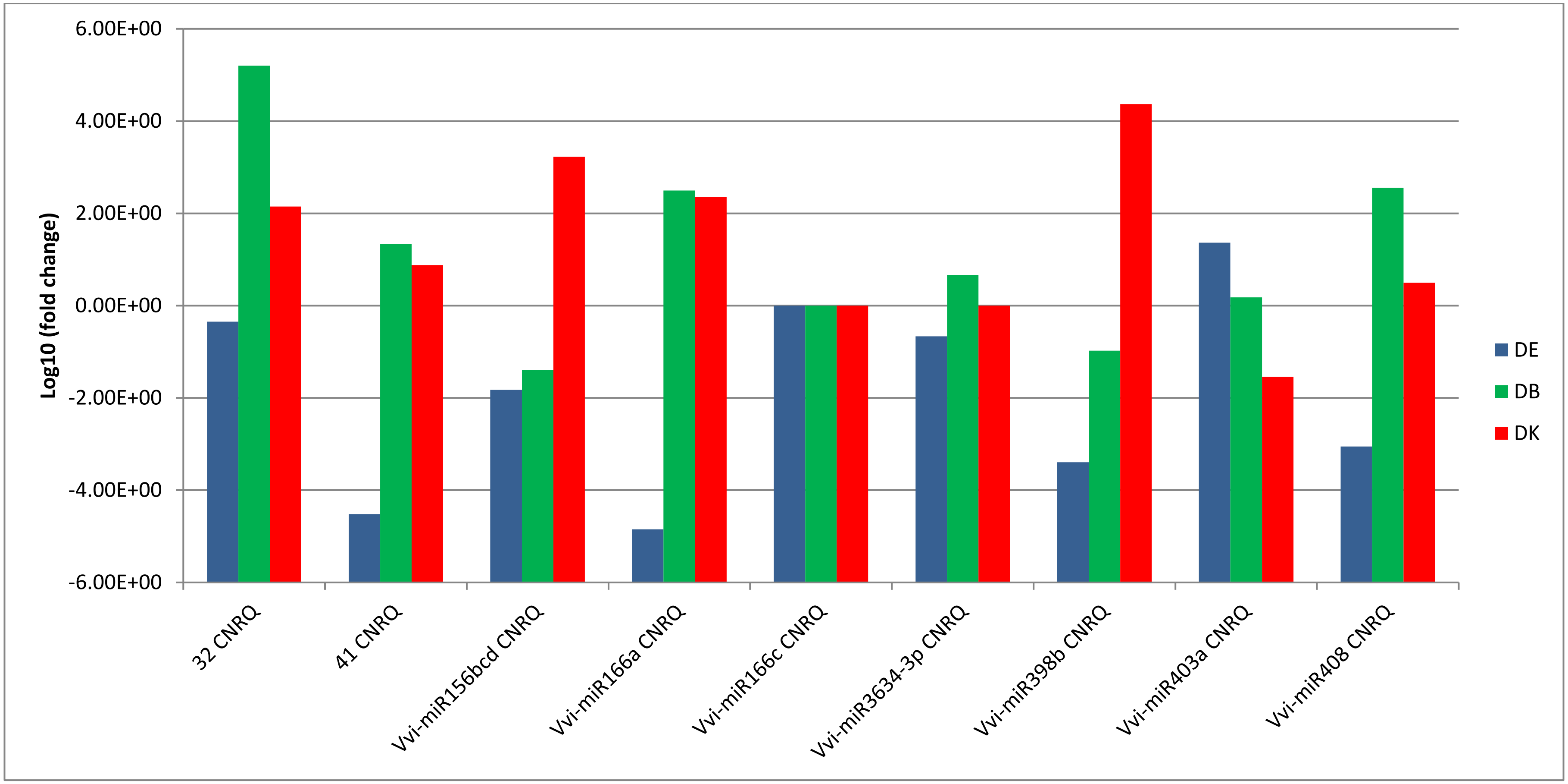

Figure S1. Relative quantification based on stem-loop RT-qPCR of differently expressed miRNAs in different variants. miRNA Vvi-miR 166c is the reference miRNA and the bars represent fold expressions compared to the reference miRNA. DE is the *Diaporthe eres* treated, DB is *Diaporthe bohemiae* treated and C is control. Relative miRNA quantification was analysed in qBasePLUS v3.2 software (Biogazelle, Ghent, Belgium).

Table S1. List of primer sequences used in the real-time RT-qPCR assay for relative quantification of target miRNAs.

Name	Primer	Sequence 5 ´- 3 ´[a]	Length (nt)	Tm (℃)[b]
**Vvi-miR156b,c,d**	stem-loop primer	GTTGGCTCTGGTGCAGGGTCCGAGGTATTCGCACCAGAGCCAACGTGCTC	50	
	specific forward primer	GTGGGTGACAGAAGAGAGT	19	51.09
**Vvi-miR166a**	stem-loop primer	GTTGGCTCTGGTGCAGGGTCCGAGGTATTCGCACCAGAGCCAACGGAATG	50	
	specific forward primer	TTGGGTCGGACCAGGCTT	18	52.6
**Vvi-miR166c**	stem-loop primer	GTTGGCTCTGGTGCAGGGTCCGAGGTATTCGCACCAGAGCCAACGGGGAA	50	
	specific forward primer	GTTTTCGGACCAGGCTTCA	19	51.09
**Vvi-miR398b**	stem-loop primer	GTTGGCTCTGGTGCAGGGTCCGAGGTATTCGCACCAGAGCCAACCAGGGG	50	
	specific forward primer	GTTGTGTGTTCTCAGGTCG	19	51.09

**Vvi-miR3634-3p**	stem-loop primer	GTTGGCTCTGGTGCAGGGTCCGAGGTATTCGCACCAGAGCCAACACGGCA	50	
	specific forward primer	GTTTTCCGACTCGCACTCA	19	51.09
**Vvi-miR403a**	stem-loop primer	GTTGGCTCTGGTGCAGGGTCCGAGGTATTCGCACCAGAGCCAACCGAGTT	50	
	specific forward primer	GGGGTTAGATTCACGCACA	19	51.09
**Vvi-miR408**	stem-loop primer	GTTGGCTCTGGTGCAGGGTCCGAGGTATTCGCACCAGAGCCAACGCCAGG	50	
	specific forward primer	GTTGATGCACTGCCTCTTC	19	51.09
**32**	stem-loop primer	GTTGGCTCTGGTGCAGGGTCCGAGGTATTCGCACCAGAGCCAACCCGACG	50	
	specific forward primer	TTGTCCCAGTCCCGAACC	18	52.6
**41**	stem-loop primer	GTTGGCTCTGGTGCAGGGTCCGAGGTATTCGCACCAGAGCCAACGGCCAG	50	
	specific forward primer	TTGTTCCGGCGATGCGCT	18	52.6

**universal reverse primer**	GTGCAGGGTCCGAGGT	16	51.1

[a] primers were designed using miRNA primer design tool (http://genomics.dote.hu:8080/mirnadesigntool/)

[b] based on Basic Tm calculation from http://www.biophp.org

Table S2. Identification of total known grapevine miRNAs in complete dataset. Data were generated by CLC Genomics WB 6.5.1, the reads were analyzed using extraction and counting of the reads. Annotation and merging of the counts were used.

Nr.	Feature ID	Expression values	Name	Resource	Exact mature 5'	Mature 5'	Unique exact mature 5'	Unique mature 5'	Exact mature 3'	Mature 3'	Unique exact mature 3'	Unique mature 3'	Exact other	Other	Total
1	MIR396b (Vitis vinifera)	8 763,00	MIR396b	Vitis vinifera	2778	8763	2778	8632	0	0	0	0	177	277	9040
2	MIR3623 (Vitis vinifera)	4 860,00	MIR3623	Vitis vinifera	2019	4860	2019	4860	47	3414	47	3414	269	576	8850
3	MIR156i (Vitis vinifera)	2 896,00	MIR156i	Vitis vinifera	716	2896	0	0	0	0	0	0	8	19	2915
4	MIR156f (Vitis vinifera)	2 894,00	MIR156f	Vitis vinifera	715	2894	0	2	0	0	0	0	3	8	2902
5	MIR156g (Vitis vinifera)	2 885,00	MIR156g	Vitis vinifera	715	2885	0	0	0	0	0	0	745	2740	5625
6	MIR397a (Vitis vinifera)	1 542,00	MIR397a	Vitis vinifera	1011	1542	1011	1542	0	0	0	0	1267	2262	3804
7	MIR396c (Vitis vinifera)	780	MIR396c	Vitis vinifera	232	780	0	188	0	0	0	0	0	40	820
8	MIR396d (Vitis vinifera)	495	MIR396d	Vitis vinifera	232	495	0	6	0	0	0	0	56	96	591
9	MIR167c (Vitis vinifera)	465	MIR167c	Vitis vinifera	340	465	340	423	0	0	0	0	115	129	594
10	MIR156d (Vitis vinifera)	394	MIR156d	Vitis vinifera	126	394	0	60	0	0	0	0	39	53	447
11	MIR168 (Vitis vinifera)	368	MIR168	Vitis vinifera	227	368	227	368	0	0	0	0	30	52	420
12	MIR156b (Vitis vinifera)	337	MIR156b	Vitis vinifera	127	337	0	0	0	0	0	0	85	98	435
13	MIR156c (Vitis vinifera)	335	MIR156c	Vitis vinifera	127	335	0	0	0	0	0	0	20	23	358
14	MIR396a (Vitis vinifera)	330	MIR396a	Vitis vinifera	62	330	62	75	0	0	0	0	12	20	350

15	MIR3633a (Vitis vinifera)	281	MIR3633a	Vitis vinifera	198	281	198	281	42	251	42	251	24	30	562
16	MIR3639 (Vitis vinifera)	263	MIR3639	Vitis vinifera	66	263	66	263	0	1	0	1	189	217	481
17	MIR167a (Vitis vinifera)	193	MIR167a	Vitis vinifera	101	193	101	153	0	0	0	0	2	2	195
18	MIR156e (Vitis vinifera)	160	MIR156e	Vitis vinifera	0	160	0	3	0	0	0	0	0	0	160
19	MIR3633b (Vitis vinifera)	148	MIR3633b	Vitis vinifera	12	148	12	148	9	99	9	99	0	1	248
20	MIR393a (Vitis vinifera)	124	MIR393a	Vitis vinifera	20	124	0	3	0	0	0	0	6	6	130
21	MIR393b (Vitis vinifera)	118	MIR393b	Vitis vinifera	20	118	0	0	0	0	0	0	0	0	118
22	MIR167b (Vitis vinifera)	81	MIR167b	Vitis vinifera	19	81	0	5	0	0	0	0	197	234	315
23	MIR167e (Vitis vinifera)	78	MIR167e	Vitis vinifera	19	78	0	0	0	0	0	0	106	113	191
24	MIR394b (Vitis vinifera)	77	MIR394b	Vitis vinifera	59	77	59	77	0	0	0	0	0	0	77
25	MIR390 (Vitis vinifera)	68	MIR390	Vitis vinifera	57	68	57	68	0	0	0	0	3	4	72
26	MIR167d (Vitis vinifera)	65	MIR167d	Vitis vinifera	19	65	0	2	0	0	0	0	1	8	73
27	MIR3626 (Vitis vinifera)	54	MIR3626	Vitis vinifera	2	54	2	54	0	9	0	9	36	47	110
28	MIR535a (Vitis vinifera)	49	MIR535a	Vitis vinifera	12	49	0	0	0	0	0	0	1	2	51
29	MIR2950 (Vitis vinifera)	49	MIR2950	Vitis vinifera	33	49	33	49	0	0	0	0	0	0	49
30	MIR3634 (Vitis vinifera)	48	MIR3634	Vitis vinifera	25	48	25	48	32797	38605	32797	38605	348	489	39142
31	MIR535b (Vitis vinifera)	47	MIR535b	Vitis vinifera	12	47	0	0	0	0	0	0	2	3	50

32	MIR535c (Vitis vinifera)	47	MIR535c	Vitis vinifera	12	47	0	0	0	0	0	0	51	73	120
33	MIR2111 (Vitis vinifera)	35	MIR2111	Vitis vinifera	29	35	29	35	3	4	3	4	0	0	39
34	MIR3636 (Vitis vinifera)	26	MIR3636	Vitis vinifera	5	26	5	26	41	96	41	96	22	26	148
35	MIR160c (Vitis vinifera)	24	MIR160c	Vitis vinifera	7	24	0	1	0	0	0	0	1	1	25
36	MIR3627 (Vitis vinifera)	22	MIR3627	Vitis vinifera	18	22	18	22	41	47	41	47	0	0	69
37	MIR160e (Vitis vinifera)	19	MIR160e	Vitis vinifera	6	19	0	0	0	0	0	0	1	2	21
38	MIR169e (Vitis vinifera)	19	MIR169e	Vitis vinifera	3	19	3	18	0	0	0	0	0	13	32
39	MIR828a (Vitis vinifera)	19	MIR828a	Vitis vinifera	0	19	0	19	0	0	0	0	5	9	28
40	MIR160d (Vitis vinifera)	16	MIR160d	Vitis vinifera	6	16	0	0	0	0	0	0	0	0	16
41	MIR3640 (Vitis vinifera)	14	MIR3640	Vitis vinifera	11	14	11	14	9	11	9	11	124	181	206
42	MIR3630 (Vitis vinifera)	11	MIR3630	Vitis vinifera	0	11	0	11	0	5	0	5	12	13	29
43	MIR164c (Vitis vinifera)	8	MIR164c	Vitis vinifera	3	8	0	1	0	0	0	0	0	0	8
44	MIR394a (Vitis vinifera)	8	MIR394a	Vitis vinifera	0	8	0	1	0	0	0	0	5	5	13
45	MIR479 (Vitis vinifera)	7	MIR479	Vitis vinifera	5	7	5	7	0	0	0	0	128	142	149
46	MIR3632 (Vitis vinifera)	7	MIR3632	Vitis vinifera	3	7	3	7	21	122	21	122	2	2	131
47	MIR169g (Vitis vinifera)	6	MIR169g	Vitis vinifera	1	6	0	5	0	0	0	0	48	230	236
48	MIR156a (Vitis	5	MIR156a	Vitis vinifera	0	5	0	1	0	0	0	0	0	0	5

	vinifera)														
49	MIR164a (Vitis vinifera)	5	MIR164a	Vitis vinifera	2	5	0	0	0	0	0	0	0	0	5
50	MIR164d (Vitis vinifera)	4	MIR164d	Vitis vinifera	3	4	0	0	0	0	0	0	1	2	6
51	MIR394c (Vitis vinifera)	4	MIR394c	Vitis vinifera	0	4	0	0	0	0	0	0	6	6	10
52	MIR3631a (Vitis vinifera)	4	MIR3631a	Vitis vinifera	2	4	0	1	0	1	0	1	0	34	39
53	MIR169v (Vitis vinifera)	3	MIR169v	Vitis vinifera	2	3	2	3	0	0	0	0	0	0	3
54	MIR3631c (Vitis vinifera)	3	MIR3631c	Vitis vinifera	1	3	0	0	0	0	0	0	0	6	9
55	MIR169d (Vitis vinifera)	2	MIR169d	Vitis vinifera	2	2	2	2	0	0	0	0	1	2	4
56	MIR160a (Vitis vinifera)	1	MIR160a	Vitis vinifera	0	1	0	0	0	0	0	0	1	1	2
57	MIR160b (Vitis vinifera)	1	MIR160b	Vitis vinifera	0	1	0	0	0	0	0	0	1	1	2
58	MIR169s (Vitis vinifera)	1	MIR169s	Vitis vinifera	0	1	0	0	0	0	0	0	0	0	1
59	MIR156h (Vitis vinifera)	1	MIR156h	Vitis vinifera	0	1	0	0	0	0	0	0	0	0	1
60	MIR169h (Vitis vinifera)	1	MIR169h	Vitis vinifera	0	1	0	0	0	0	0	0	0	0	1
61	MIR169l (Vitis vinifera)	1	MIR169l	Vitis vinifera	0	1	0	0	0	0	0	0	0	0	1
62	MIR169w (Vitis vinifera)	1	MIR169w	Vitis vinifera	0	1	0	0	0	0	0	0	0	0	1
63	MIR169x (Vitis vinifera)	1	MIR169x	Vitis vinifera	0	1	0	0	0	0	0	0	0	0	1
64	MIR3624 (Vitis vinifera)	1	MIR3624	Vitis vinifera	1	1	1	1	174	192	174	192	83	109	302
65	MIR3631b (Vitis	1	MIR3631b	Vitis vinifera	1	1	0	0	0	5	0	5	8	43	49

	vinifera)														
66	MIR3637 (Vitis vinifera)	1	MIR3637	Vitis vinifera	1	1	1	1	2	5	2	5	11	18	24
67	MIR159a (Vitis vinifera)	0	MIR159a	Vitis vinifera	0	0	0	0	0	6	0	0	1	1	7
68	MIR159b (Vitis vinifera)	0	MIR159b	Vitis vinifera	0	0	0	0	0	7	0	0	0	0	7
69	MIR159c (Vitis vinifera)	0	MIR159c	Vitis vinifera	0	0	0	0	4282	5418	4282	5416	499	553	5971
70	MIR162 (Vitis vinifera)	0	MIR162	Vitis vinifera	0	0	0	0	2599	3044	2599	3044	4	24	3068
71	MIR166a (Vitis vinifera)	0	MIR166a	Vitis vinifera	0	0	0	0	1793	39684	0	37571	14	46	39730
72	MIR166b (Vitis vinifera)	0	MIR166b	Vitis vinifera	0	0	0	0	1794	2998	0	898	74	121	3119
73	MIR166c (Vitis vinifera)	0	MIR166c	Vitis vinifera	0	0	0	0	37607	39576	0	0	577	625	40201
74	MIR166d (Vitis vinifera)	0	MIR166d	Vitis vinifera	0	0	0	0	37608	39761	0	10	108	122	39883
75	MIR166e (Vitis vinifera)	0	MIR166e	Vitis vinifera	0	0	0	0	37607	39587	0	0	572	622	40209
76	MIR166f (Vitis vinifera)	0	MIR166f	Vitis vinifera	0	0	0	0	37607	40541	0	1	36	48	40589
77	MIR166g (Vitis vinifera)	0	MIR166g	Vitis vinifera	0	0	0	0	37607	41080	0	559	18	31	41111
78	MIR166h (Vitis vinifera)	0	MIR166h	Vitis vinifera	0	0	0	0	37607	39695	0	54	93	121	39816
79	MIR169a (Vitis vinifera)	0	MIR169a	Vitis vinifera	0	0	0	0	0	0	0	0	1	2	2
80	MIR169c (Vitis vinifera)	0	MIR169c	Vitis vinifera	0	0	0	0	0	0	0	0	2	7	7
81	MIR169j (Vitis vinifera)	0	MIR169j	Vitis vinifera	0	0	0	0	0	0	0	0	4	4	4

82	MIR169k (Vitis vinifera)	0	MIR169k	Vitis vinifera	0	0	0	0	0	0	0	0	5	5	5
83	MIR169r (Vitis vinifera)	0	MIR169r	Vitis vinifera	0	0	0	0	0	0	0	0	4	5	5
84	MIR169t (Vitis vinifera)	0	MIR169t	Vitis vinifera	0	0	0	0	0	0	0	0	5	6	6
85	MIR169u (Vitis vinifera)	0	MIR169u	Vitis vinifera	0	0	0	0	0	0	0	0	22	30	30
86	MIR171a (Vitis vinifera)	0	MIR171a	Vitis vinifera	0	0	0	0	4	7	0	0	2	25	32
87	MIR171b (Vitis vinifera)	0	MIR171b	Vitis vinifera	0	0	0	0	1	1	1	1	33	34	35
88	MIR171c (Vitis vinifera)	0	MIR171c	Vitis vinifera	0	0	0	0	5	7	0	0	1	1	8
89	MIR171d (Vitis vinifera)	0	MIR171d	Vitis vinifera	0	0	0	0	5	8	0	0	2	4	12
90	MIR171e (Vitis vinifera)	0	MIR171e	Vitis vinifera	0	0	0	0	0	0	0	0	1	1	1
91	MIR171f (Vitis vinifera)	0	MIR171f	Vitis vinifera	0	0	0	0	2	4	2	4	2	2	6
92	MIR171i (Vitis vinifera)	0	MIR171i	Vitis vinifera	0	0	0	0	5	7	0	0	0	22	29
93	MIR172c (Vitis vinifera)	0	MIR172c	Vitis vinifera	0	0	0	0	0	0	0	0	0	1	1
94	MIR172d (Vitis vinifera)	0	MIR172d	Vitis vinifera	0	0	0	0	0	1	0	1	1	5	6
95	MIR319b (Vitis vinifera)	0	MIR319b	Vitis vinifera	0	0	0	0	75	634	0	1	3	6	640
96	MIR319c (Vitis vinifera)	0	MIR319c	Vitis vinifera	0	0	0	0	75	639	0	0	19	23	662
97	MIR319f (Vitis vinifera)	0	MIR319f	Vitis vinifera	0	0	0	0	74	644	0	0	13	16	660
98	MIR319g (Vitis vinifera)	0	MIR319g	Vitis vinifera	0	0	0	0	3	257	3	3	0	0	257

99	MIR395a (Vitis vinifera)	0	MIR395a	Vitis vinifera	0	0	0	0	1	1	0	0	0	0	1
100	MIR395b (Vitis vinifera)	0	MIR395b	Vitis vinifera	0	0	0	0	0	0	0	0	0	0	0
101	MIR395c (Vitis vinifera)	0	MIR395c	Vitis vinifera	0	0	0	0	0	0	0	0	1	1	1
102	MIR395e (Vitis vinifera)	0	MIR395e	Vitis vinifera	0	0	0	0	0	1	0	0	2	2	3
103	MIR395h (Vitis vinifera)	0	MIR395h	Vitis vinifera	0	0	0	0	1	1	0	0	1	1	2
104	MIR398a (Vitis vinifera)	0	MIR398a	Vitis vinifera	0	0	0	0	6	104	6	25	9	10	114
105	MIR399a (Vitis vinifera)	0	MIR399a	Vitis vinifera	0	0	0	0	47	77	0	0	20	23	100
106	MIR399b (Vitis vinifera)	0	MIR399b	Vitis vinifera	0	0	0	0	41	45	0	1	36	37	82
107	MIR399e (Vitis vinifera)	0	MIR399e	Vitis vinifera	0	0	0	0	7	24	7	10	1	1	25
108	MIR399g (Vitis vinifera)	0	MIR399g	Vitis vinifera	0	0	0	0	51	72	51	59	10	12	84
109	MIR399h (Vitis vinifera)	0	MIR399h	Vitis vinifera	0	0	0	0	47	77	0	0	7	9	86
110	MIR408 (Vitis vinifera)	0	MIR408	Vitis vinifera	0	0	0	0	6610	18470	6610	18470	1101	1750	20220
111	MIR169b (Vitis vinifera)	0	MIR169b	Vitis vinifera	0	0	0	0	0	0	0	0	1	1	1
112	MIR169n (Vitis vinifera)	0	MIR169n	Vitis vinifera	0	0	0	0	0	0	0	0	2	2	2
113	MIR169q (Vitis vinifera)	0	MIR169q	Vitis vinifera	0	0	0	0	0	0	0	0	2	3	3
114	MIR171g (Vitis vinifera)	0	MIR171g	Vitis vinifera	0	0	0	0	0	1	0	1	0	0	1
115	MIR319e (Vitis	0	MIR319e	Vitis vinifera	0	0	0	0	23	52	23	47	0	0	52

	vinifera)														
116	MIR398b (Vitis vinifera)	0	MIR398b	Vitis vinifera	0	0	0	0	37448	40188	0	0	163	181	40369
117	MIR398c (Vitis vinifera)	0	MIR398c	Vitis vinifera	0	0	0	0	37448	40195	0	0	198	228	40423
118	MIR399c (Vitis vinifera)	0	MIR399c	Vitis vinifera	0	0	0	0	41	42	0	0	1	1	43
119	MIR399d (Vitis vinifera)	0	MIR399d	Vitis vinifera	0	0	0	0	0	1	0	1	1	1	2
120	MIR399i (Vitis vinifera)	0	MIR399i	Vitis vinifera	0	0	0	0	245	257	245	257	11	13	270
121	MIR403a (Vitis vinifera)	0	MIR403a	Vitis vinifera	0	0	0	0	478	685	0	0	3	6	691
122	MIR403b (Vitis vinifera)	0	MIR403b	Vitis vinifera	0	0	0	0	479	665	0	0	7	8	673
123	MIR403c (Vitis vinifera)	0	MIR403c	Vitis vinifera	0	0	0	0	478	675	0	0	1	5	680
124	MIR403d (Vitis vinifera)	0	MIR403d	Vitis vinifera	0	0	0	0	479	655	0	0	2	4	659
125	MIR403e (Vitis vinifera)	0	MIR403e	Vitis vinifera	0	0	0	0	479	670	0	11	2	2	672
126	MIR403f (Vitis vinifera)	0	MIR403f	Vitis vinifera	0	0	0	0	478	682	0	5	7	14	696
127	MIR477a (Vitis vinifera)	0	MIR477a	Vitis vinifera	0	0	0	0	0	0	0	0	2	7	7
128	MIR482 (Vitis vinifera)	0	MIR482	Vitis vinifera	0	0	0	0	615	694	615	694	786	860	1554
129	MIR845a (Vitis vinifera)	0	MIR845a	Vitis vinifera	0	0	0	0	0	0	0	0	3	7	7
130	MIR845b (Vitis vinifera)	0	MIR845b	Vitis vinifera	0	0	0	0	0	0	0	0	3	5	5
131	MIR845d (Vitis vinifera)	0	MIR845d	Vitis vinifera	0	0	0	0	0	0	0	0	0	1	1
132	MIR3625 (Vitis	0	MIR3625	Vitis vinifera	0	0	0	0	0	1	0	1	0	1	2

	vinifera)													
133	MIR3629a (Vitis vinifera)	0	MIR3629a	Vitis vinifera	0	0	0	0	0	0	0	0	1	1	1
134	MIR3631d (Vitis vinifera)	0	MIR3631d	Vitis vinifera	0	0	0	0	0	0	0	0	0	8	8
135	MIR3635 (Vitis vinifera)	0	MIR3635	Vitis vinifera	0	0	0	0	1	4	1	4	1	1	5
136	MIR171j (Vitis vinifera)	0	MIR171j	Vitis vinifera	0	0	0	0	5	5	0	0	2	2	7

Table S3. Representation of known miRNAs in the treatments DE, DB and C.

	**DE**	**DB**	**C**
**Name**	**Total**	**Total**	**Total**
**MIR156a**	0	0	4
**MIR156b**	6	7	410
**MIR156c**	3	9	352
**MIR156d**	5	9	428
**MIR156e**	0	1	160
**MIR156f**	9	111	2709
**MIR156g**	18	203	5262
**MIR156h**	0	0	1
**MIR156i**	7	113	2726
**MIR159a**	2	2	2
**MIR159b**	0	0	4
**MIR159c**	25	364	5368
**MIR160c**	2	5	14
**MIR160d**	0	3	16
**MIR160e**	2	1	18
**MIR162**	17	365	2314
**MIR164a**	0	1	4
**MIR164b**	0	0	1

**MIR164c**	0	0	5
**MIR164d**	2	0	4
**MIR166a**	59	1481	36362
**MIR166b**	12	181	2837
**MIR166c**	21	880	38480
**MIR166d**	20	889	38201
**MIR166e**	20	870	38500
**MIR166f**	15	898	38864
**MIR166g**	16	921	39352
**MIR166h**	28	913	38059
**MIR167a**	2	4	188
**MIR167b**	6	13	288
**MIR167c**	1	26	554
**MIR167d**	1	3	67
**MIR167e**	2	11	171
**MIR168**	13	30	297
**MIR169a**	0	2	3
**MIR169b**	1	2	1
**MIR169c**	0	2	7
**MIR169d**	0	0	3
**MIR169e**	1	2	29
**MIR169f**	0	0	1
**MIR169g**	2	5	224
**MIR169j**	0	0	4
**MIR169k**	0	0	5
**MIR169l**	0	0	1
**MIR169n**	0	0	3
**MIR169q**	0	0	2
**MIR169r**	1	1	3
**MIR169s**	0	0	1
**MIR169t**	0	0	7

**MIR169u**	0	1	28
**MIR169v**	0	0	3
**MIR169w**	0	0	1
**MIR169x**	0	0	1
**MIR171a**	1	0	33
**MIR171b**	1	0	33
**MIR171c**	0	0	5
**MIR171d**	1	1	8
**MIR171e**	0	0	1
**MIR171f**	0	0	5
**MIR171g**	0	0	1
**MIR171i**	0	2	27
**MIR171j**	0	1	7
**MIR172a**	0	0	3
**MIR172b**	0	1	4
**MIR172d**	0	1	1
**MIR2111**	0	1	36
**MIR2950**	0	5	40
**MIR319b**	3	22	593
**MIR319c**	3	21	612
**MIR319e**	7	8	42
**MIR319f**	7	21	604
**MIR319g**	0	10	231
**MIR3623**	0	1392	6930
**MIR3624**	0	65	224
**MIR3625**	0	11	2
**MIR3626**	0	11	88
**MIR3627**	0	12	56
**MIR3629a**	0	11	1
**MIR3630**	0	11	10

**MIR3631a**	0	4	71
**MIR3631b**	0	6	57
**MIR3631c**	0	1	8
**MIR3631d**	0	0	10
**MIR3632**	0	25	77
**MIR3633a**	0	74	452
**MIR3633b**	0	6	223
**MIR3634**	0	4060	33140
**MIR3635**	0	1	3
**MIR3636**	0	4	138
**MIR3637**	0	2	19
**MIR3639**	0	20	446
**MIR3640**	0	9	186
**MIR390**	2	3	66
**MIR393a**	0	24	92
**MIR393b**	2	18	88
**MIR394a**	2	4	5
**MIR394b**	2	21	34
**MIR394c**	0	0	7
**MIR395c**	1	4	1
**MIR395f**	0	0	1
**MIR395k**	0	0	2
**MIR395l**	3	20	0
**MIR395m**	24	477	0
**MIR396a**	3	20	320
**MIR396b**	24	477	8288
**MIR396c**	0	75	703
**MIR396d**	1	59	495
**MIR397a**	0	165	3375
**MIR398a**	3	15	95
**MIR398b**	0	1487	38053

**MIR398c**	0	1473	38111
**MIR399a**	1	9	88
**MIR399b**	2	3	75
**MIR399c**	0	1	39
**MIR399d**	0	1	1
**MIR399e**	2	3	18
**MIR399g**	0	10	74
**MIR399h**	2	8	77
**MIR399i**	27	17	239
**MIR403a**	2	66	592
**MIR403b**	2	65	582
**MIR403c**	0	63	589
**MIR403d**	0	68	566
**MIR403e**	2	66	564
**MIR403f**	7	68	602
**MIR408**	27	803	18894
**MIR477a**	0	0	7
**MIR479**	2	9	136
**MIR482**	0	0	1341
**MIR535a**	2	115	60
**MIR535b**	0	2	61
**MIR535c**	0	2	89
**MIR828a**	3	0	15
**MIR845a**	0	0	8
**MIR845b**	0	0	10
**MIR845e**	0	0	2

Table S4. Representation of new miRNAs in the treatments DE, DB and C.

miRNA name	miRNA sequence	DE	DB	C
**2**	CCCAGUCCCGAACCCGUCGGC	5202	1625	44959
**3**	AGUUACUAAUUCAUGAUCUGGC	0	2075	36015
**5**	CCAGUCCCGAACCCGUCGGC	1484	0	17694
**6**	UCUCGGACCAGGCUUCAUUCC	0	602	15662
**8**	GGUGGCUGUAGUUUAGUGGU	10597	767	4692
**9**	CGGUGGACUGCUCGAGCUGC	2353	687	3863
**10**	CUAACAGACCGGUAGACUUGAAC	0	0	3057
**12**	CCCAGUCCCGAACCCGUCGGCU	0	1625	2726
**13**	GCGCCUGUAGCUCAGUGGA	1425	4407	2476
**14**	UUCAUGGACGUUGAUAAGAUCCU	0	0	2434
**15**	UAACAGACCGGUAGACUUGAAC	0	0	2204
**16**	UGCACUGCCUCUUCCCUGGCU	0	0	2084
**17**	CCUAACAGACCGGUAGACUUGAAC	0	0	2025
**18**	UCCUAACAGACCGGUAGACUUGAAC	0	0	2007
**19**	UCCUAACAGACCGGUAGACUUGAAC	0	0	1970
**20**	UUAGAUGAUCAUCAACAAACU	0	985	1947
**21**	CAGACCGGUAGACUUGAAC	0	0	1899
**23**	ACAGACCGGUAGACUUGAAC	0	0	1859
**24**	UUCCACAGCUUUCUUGAACUU	0	0	1803
**25**	AACAGACCGGUAGACUUGAAC	0	0	1757
**26**	CCGGCGAUGCGCUCCUGGCC	12320	4115	1600
**27**	CAGUCCCGAACCCGUCGGC	0	593	1590
**28**	UGUUGAGCUCACCUUGUACCC	0	0	1554
**30**	CGGUGGACUGCUCGAGCUGCU	1782	800	1499
**31**	GUUGAGCUCACCUUGUACCCA	990	0	1455
**32**	CCCAGUCCCGAACCCGUCGG	0	22014	1434
**33**	UGAAGGUCCAAGGCCGAGGCU	0	0	1403
**34**	GGGAUGGGUCGACCGGUCC	1289	961	1397
**35**	UCGGAUAAAGGGUUAUACAUC	0	0	1344
**36**	UGCACUGCCUCUUCCCUGGC	0	0	1222
**37**	CUGGAUUAUGACUGAACGCCU	0	0	1216
**38**	UUCCACAGCUUUCUUGAACU	0	0	1195
**40**	AGUUACUAAUUCAUGAUCUGGCC	0	0	1190

**41**	CCGGCGAUGCGCUCCUGGCC	12320	22014	1600
**42**	CCGGCGAUGCGCUCCUGGCCU	7767	2381	927
**43**	ACCGGCGAUGCGCUCCUGGCCU	3561	1746	936
**44**	GCCCGUGGAGACGUCGUCGCCUCG	3273	1259	0
**45**	CGCCGUCCGAAUUGUAGUCUGGA	3262	1074	0
**46**	UCGGGUUAACAUUCCUGAACCGGGA	3259	855	0
**47**	CGGUGGACUGCUCGAGCUGCU	1782	687	0
**48**	CCAGUCCCGAACCCGUCGGC	1484	8369	17694
